# Retrospective analysis of pathological changes in the maxillary sinus with CBCT

**DOI:** 10.1038/s41598-024-66527-7

**Published:** 2024-07-05

**Authors:** Mehmet Emin Dogan, Nurbanu Uluısık, Semahat Doğru Yuvarlakbaş

**Affiliations:** 1 Faculty of Dentistry, Department of Dentomaxillofacial Radiology, Harran University, Sanliurfa, Turkey; 2 Faculty of Medicine, Department of Anatomy, Harran University, Sanliurfa, Turkey

**Keywords:** Maxillary sinus, Cone beam computed tomography, Anatomical variation, Pathology, Mucosal thickening, Diseases, Medical research

## Abstract

The aim of this study is to examine the frequency of maxillary sinus pathologies and their relationship with each other using cone beam computed tomography (CBCT) and to make a new grade according to the number of affected walls. 1000 maxillary sinuses of 500 patients with CBCT images were included in the study. Anatomical variations and pathological formations of the maxillary sinuses were examined. Images were evaluated for the presence of flat, polypoidal, partial and generalized mucosal thickening, partial and total opacification, polyps and mucous retention cysts. Maxillary sinus pathologies were graded according to the number of walls affected. In the examined CBCT images, no pathology was found in 54.2% of the maxillary sinuses, while pathology was observed in 45.8%. The most common sinus pathologies were mucous retention cyst (12.3%) and polypoidal thickening (12.2%). While pneumatization, ostium obstruction, and the presence of sinus-related roots were associated with sinus pathology, no relationship was found with nasal septum deviation and the presence of septa. Before dental implant and sinus surgery applications, the presence of sinus pathologies and their relationship with anatomical variations can be evaluated with CBCT, a three-dimensional technique, and complications such as sinus membrane perforation, infection, failure to break the bone window due to the presence of antral septa, graft loss and oroantral fistula formation can be reduced.

## Introduction

The paranasal sinuses are air cavities located in the bones of the skull surrounding the nasal cavity. The importance of the paranasal sinuses has increased because they are covered with mucosa and connected to the nasal cavity. Being lined with mucosa and connected to the nasal cavity, these sinuses are associated with many important functions, including respiratory infections, allergic reactions, and mucus drainage. Therefore, the health of the paranasal sinuses is critical to overall nasal and respiratory health. The most obvious function of the paranasal sinuses, which are assumed to have many functions such as reducing skull weight, resonance of sound, absorption of trauma, heat insulation, adjustment of intranasal pressure and many others, is ventilation and drainage^1–3^. Paranasal sinuses are complex structures that are difficult to examine because they vary in size, shape and volume^4,5^.

The maxillary sinuses are the most interesting region among the paranasal sinuses due to their large volume, neighborhood and variations. The maxillary sinuses begin to develop between the second and third months of pregnancy and enlarge with the eruption of permanent teeth. Their inner walls are covered with Schneiderian membrane. The thickness of the Schneiderian membrane varies between 0.13 and 0.5 mm, but various etiologic conditions may cause an increase in membrane thickness^6,7^.

Inflammatory lesions and cystic conditions are among the most widespread maxillary sinus pathologies in the literature^8^. Maxillary sinus retention cysts (MSRCs) are the most common in this group. Although most of them are small and asymptomatic, they may increase in size and obstruct the ostium of the maxillary sinus in some cases^9^. Various inflammatory conditions may also cause opacification by thickening the mucosa and increasing the opacity in the region. Thus, the amount of air decreases and the osteomeatal opening may be narrowed or obstructed^10^.

Cystic lesions in the maxillary sinus are also among the most common conditions. Retention cysts occur when the seromucinous gland canals in the sinus mucosa become blocked. These are lesions that cause symptoms such as headache, nasal congestion, and runny nose^10^. Appropriate imaging is crucial for diagnosis.

With the advancement of technology, imaging methods have also improved. In recent years, CBCT has been preferred because it is inexpensive and minimizes radiation exposure^11^. In addition to understanding the complex anatomical structure of the maxillary sinuses, CBCT is the best imaging method that can be used in this field, providing three-dimensional (3D) imaging to identify and measure pathologic conditions and variations in the region. The fact that it provides detailed imaging especially in bone tissue facilitates both surgical interventions and the detection of variations^5^. The maxillary sinus is a significant anatomical structure in the clinical practice of dentistry. This structure should be evaluated in terms of pathologic lesions and variations during implant planning, especially in cases of maxillary posterior edentulism.

In this study, we aimed to contribute to the literature by analysing the prevalence and interrelationships of maxillary sinus pathologies using CBCT and by making a new grading according to the number of affected walls.

## Material and method

Before starting the study, approval was received from the Harran University Clinical Research Ethics Committee (approval number: 23.09.12). Additionally, this study was conducted in accordance with the principles defined in the Declaration of Helsinki. In retrospective studies, the ethics committee waived the informed consent form. In this study, the images obtained with the Castellini X Radius Trioplus (imola, ITALY) device in 2022–2023 in the archive of the Dentomaxillofacial Radiology department were used. Sample size was calculated using the G Power 3.1 program. The minimum sample size was found to be 220 when α = 0.05 and test power 1-β = 0.95. Multiplanar images were obtained with a voxel size of 0.3 mm^3^, slice thickness of 1 mm, and 13 × 16 FOV (field of view).

Inclusion criteria were CBCT images without any distortion and artifacts with clear visualization of the maxillary sinuses.

Exclusion criteria: Images with odontogenic cysts, tumors, facial growth defects, trauma affecting the midface and maxillary sinuses were excluded. Images containing diseases affecting the bone such as Paget, fibrous dysplasia and osteopetrosis were excluded. Additionally, those with a history of maxillofacial surgery were excluded from the study.

### Image analysis

IRYS version 15.1 was used to analyze the images. CBCT images were evaluated on the right and left sides for the presence of polyps, mucosal thickening (flat, polypoid, partial and generalized), opacification (partial and total), and mucous retention cysts. All images were analyzed by a dentomaxillofacial radiologist (MED) with 5 years of experience. Undecided cases were excluded. Observation and examination were performed in all planes to avoid missing inflammatory pathologies of the maxillary sinus as follows.

#### Normal

**Figure 1:**
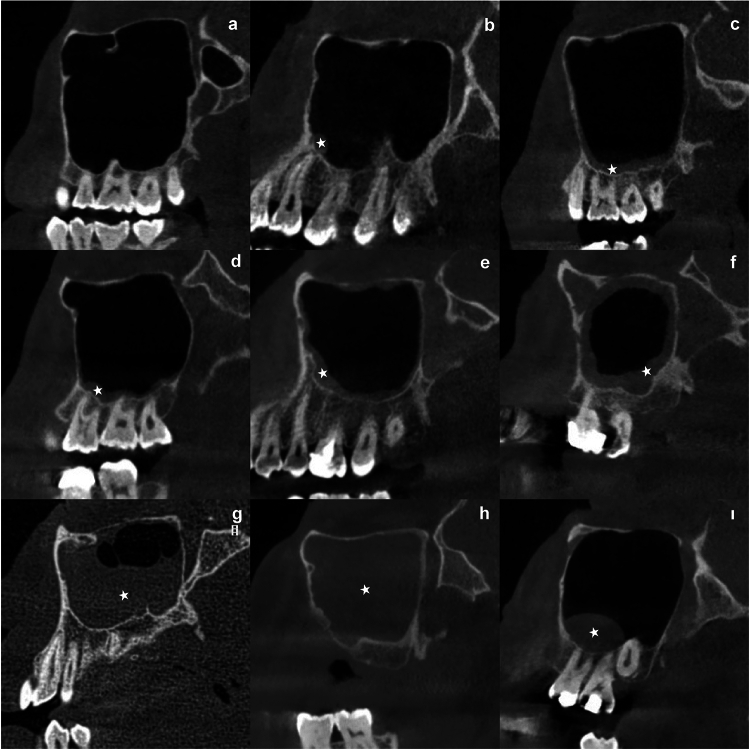
(**a**) Normal sinus, (**b**) Polyp, (**c**) Flat mucosal thickening, (**d**) Polypoidal mucosal thickening, (**e**)Partial mucosal thickening, (**f**) Generalized mucosal thickening, (**g**)Partial opacification, (**h**) Total opacification, (**ı**):Mucous retention cyst.

No pathologic changes in the mucosa and internal structure of the maxillary sinus (Fig. 1a).

#### Polyps

These are rounded and stalked folds of tissue with smooth borders, originating from thickened maxillary sinus mucosa with chronic inflammation, isolated in a single area or present in different areas within the sinus^12^ (Fig. 1b).

#### Mucosal thickening

These are thickenings in the form of a diffuse radiopaque line parallel to the maxillary sinus wall, with clear borders and non-cortical^13^. If the mucosal thickness is 1 mm or more, it is considered as mucosal thickening. Mucosal thickening of more than 2 mm is considered of sinus inflammation ^14^. It was classified as flat and polypoidal according to the shape of thickening of the mucosa, and as partial and generalized according to the region affected. (Fig. 1c,d, e, f).

#### Partial and total opacification

Diffuse opacification in which more than three walls of the maxillary sinus are affected is called partial opacification, while opacification that fills the entire sinus cavity and has no air space is called total opacification^15^ (Fig. 1 g,h).

#### Mucous retention cyst

It is formed by the secretions accumulated as a result of the blockage of the secretory ducts of the seromucinous glands in the maxillary sinus mucosa. It is homogeneous, convex, well-circumscribed, dome-shaped, radiopaque lesions not surrounded by cortical bone^2,13^ (Fig. 1 ı).

Maxillary sinus pathologies were graded according to the number of walls affected as follows.

Normal: No affected wall.

Grade 1: single wall affected.

Grade 2: affected both walls.

Grade 3: three walls affected.

Grade 4: affected four walls and above.

#### Pneumatization

The relationship of the posterior tooth roots present in the dental arch with the maxillary sinus floor was investigated and if the sinus floor is displaced between the tooth roots, pneumatization was stated to be present. In addition, the presence of pneumatization in patients with no posterior teeth was recorded when the distance between the nasal floor and the maxillary sinus floor was more than 5 mm^16^.

#### Relationship between sinus and tooth root

The topographic relationship between the maxillary sinus and each apex of the upper posterior teeth was evaluated. The relationship of the root to the maxillary sinus was determined as follows: roots in the maxillary sinus and roots outside the maxillary sinus (apexes in contact or not with the borders of the maxillary sinus cortex)^17^.

### Statistical analysis

SPSS 25 (IBM Armonk, NY, USA) package program was used in statistical analysis. Descriptive statistics were used to calculate the number, percentage, mean and standard deviation values of the parameters. Chi-square test was used in pairwise comparisons. P < 0.05 was accepted as importance level.

## Results

Data from 1000 maxillary sinuses were evaluated in 500 CBCT patients, 222 (44.4%) males and 278 (55.6%) females aged between 17 and 91 years with a mean age of 40.69 ± 15.88 years. 542 (54.2%) maxillary sinuses were free of pathology while 458 (45.8%) showed pathologic changes. As shown in Table 1, the most widespread of these maxillary sinus pathologies was mucous retention cyst 12.3%, followed by polypoidal thickening 12.2%, polyp 6.6%, flat thickening 4.7%, partial opacification 4.2%, generalized thickening 3.4%, partial thickening 1.5% and total opacification 0.9%. Pneumatization (66.0%) and septum deviation (54.1%) were the most common variations. When the relationship between the affected walls and pathology was examined, it was seen that the most affected wall was inferior with 20.6% (p < 0.001). A statistically significant correlation was found between patients with ostium obstruction and maxillary sinus pathologies (p < 0.05).

**Table 1:** Distribution of variations and pathologies that may affect the maxillary sinus.

		N	(%)
Sinus pathology	Existence	458	(45.8%)
	Nonexistence	542	(54.2%)
Mucosal thickening		218	(21.8%)
	Flat thickening	47	(4.7%)
	Polypoidal thickening	122	( 12.2%)
	Partial thickening	15	(1.5%)
	Generalized thickening	34	(3.4%)
Opacification		51	(5.1%)
	Partial opacification	42	(4.2%)
	Total opacification	9	(0.9%)
Polyp		66	(6.6%)
Mucous retention cyst		123	(12.3%)
Affected side			
	Right	501	(50.1%)
	Left	499	(49.9%)
Septa in the sinus			
	Existence	370	(37.0%)
	Nonexistence	630	(63.0%)
Ostium obstruction			
	Existence	106	(10.6%)
	Nonexistence	894	(89.4%)
Septum deviation			
	Existence	541	(54.1%)
	Nonexistence	449	(45.9%)
Relationship between sinus and tooth root			
	Existence	169	(16.9%)
	Nonexistence	831	(83.1%)
Pneumatization			
	Existence	660	(66.0%)
	Nonexistence	340	(34.0%)

Especially in patients with partial opacification, ostium obstruction was observed more frequently. When the impact of nasal septum deviation on sinus pathology was analyzed, no statistically significant difference was found (p > 0.05). The absence of any pathology in patients without a tooth root associated with the maxillary sinus was found to be high and statistically significant (p < 0.05). There was no correlation between the presence of septa in the sinus and sinus pathologies (p > 0.05). However, when the pathological sinus changes observed in the maxillary sinuses with pneumatization were examined, the mucous retention cyst was found to be 9.9% more common than all other pathologies and there was a statistically significant relationship (p < 0.05) (Table 2). When the presence of right and left side maxillary sinus pathologies was analyzed comparatively, no statistically significant difference was found (p > 0.05). When the pathological findings of the maxillary sinus were categorized according to the number of walls they affected, the most common pathology was Grade 1 and the most common pathology found at this grade was mucous retention cyst 9.1% and statistically significant (p < 0.05) (Table 3). In addition, sinus pathologies were most commonly observed in the inferior wall and there was a statistically significant relationship (p < 0.05).

**Table 2:** Relationship between variations and pathologies that may affect the sinus.

	Sinus pathology	Normal	Polypoidal thickening	Straight thickening	Partial thickening	Generalized thickening	Partial opacification	Total opacification	Polyp	Mucous retention cyst	P value
Ostium obstruction	Existence	7 (0.7%)	11 (1.1%)	4 (0.4%)	3 (0.3%)	23 (2.3%)	33 (3.3%)	9 (0.9%)	3 (0.3%)	13 (1.3%)	0.000*
	Nonexistence	535 (53.5%)	111 (11.1%)	43 (4.3%)	12 (1.2%)	11 (1.1%)	9 (0.9%)	0 (0%)	63 (6.3%)	110 (11.0%)	
Nasal septum deviation	Existence	276 (27.6%)	67 (6.7%)	24 (2.4%)	10 (1.0%)	20 (2.0%)	29 (2.9%)	7 (0.7%)	31 (3.1%)	77 (7.7%)	0.072
	Nonexistence	266 (26.6%)	55 (5.5%)	23 (2.3%)	5 (0.5%)	14 (1.4%)	13 (1.3%)	2 (0.2%)	35 (3.5%)	46 (4.6%)	
Relationship between sinus and tooth root	Existence	48 (4.8%)	31 (3.1%)	18 (1.8%)	6 (0.6%)	6 (0.6%)	9 (0.9%)	2 (0.2%)	14 (1.4%)	35 (3.5%)	0.000*
	Nonexistence	494 (49.4%)	91 (9.1%)	29 (2.9%)	9 (0.9%)	28 (2.8%)	33 (3.3%)	7 (0.7%)	52 (5.2%)	88 (8.8%)	
Septa in the sinus	Existence	196 (19.6%)	47 (4.7%)	22 (2.2%)	7 (0.7%)	12 (1.2%)	8 (0.8%)	2 (0.2%)	32 (3.2%)	44 (4.4%)	0.101
	Nonexistence	346 (34.6%)	75 (7.5%)	25 (2.5%)	8 (0.8%)	22 (2.2%)	34 (3.4%)	7 (0.7%)	34 (3.4%)	79 (7.9%)	
Pneumatization	Existence	318 (31.8%)	90 (9.0%)	34 (3.4%)	11 (1.1%)	20 (2.0%)	29 (2.9%)	9 (0.9%)	50 (5.0%)	99 (9.9%)	0.000*
	Nonexistence	224 (22.4%)	32 (3.2%)	13 (1.3%)	4 (0.4%)	14 (1.4%)	13 (1.3%)	0 (0%)	16 (1.6%)	24 (2.4%)	

*p < 0.05.

**Table 3:** Grading of maxillary sinus pathologies according to the number of walls they affect.

Influenced by wall	Sinus Pathologies								
	Polypoidal thickening	Straight thickening	Partial thickening	Generalized thickening	Partial opacification	Total opacification	Polyp	Mucous retention cyst	P
Normal	0	0	0	0	0	0	0	0	0.000*
Grade 1	85 (8.5%)	46 (4.6%)	6 (0.6%)	1 (0.1%)	0	0	61 (6.1%)	91 (9.1%)	
Grade 2	29 (2.9%)	1 (0.1%)	8 (0.8%)	0	0	0	4 (0.4%)	24 (2.4%)	
Grade 3	8 (0.8%)	0	1 (0.1%)	0	0	0	1 (0.1%)	8 (0.8%)	
Grade 4	0	0	0	33 (3.3%)	42 (4.2%)	9 (0.9%)	0	0	
Total	122 (12.2%)	47 (4.7%)	15 (1.5%)	34 (3.4%)	42 (4.2%)	9 (0.9%)	66 (6.6%)	123 (12.3%)	

*p < 0.05.

## Discussion

In studies conducted in various populations, maxillary sinus abnormalities were found to be 14.3–82%^18^. In the population we examined, maxillary sinus pathologies were found to be 45.8% in parallel with these studies.

In this study, pneumatization was the most common maxillary sinus variation in accordance with the literature^19,20^. Although the age range of the studied population was similar to the study of Sanchez perez et al.^19^, ıt has been suggested that tooth loss and aging increase the likelihood of the presence of pneumatization. Maxillary sinus pneumatization may complicate implant planning and worsen the problem of bone loss caused by atrophy of the maxilla to the extent that alveolar bone remains a few millimeters^21^. The maxillary sinus septa are cortical bone barriers that separate the sinus into multiple compartments. They may be formed following maxillary development (primary), or they may be present as bony protrusions (secondary) between alveolar crest resorption, which increases with tooth loss, and progressive sinus pneumatization^22^.

The presence of septa complicates lateral window opening and sinus wall inversion in sinus floor elevation surgery^23^. The presence of maxillary sinus septa was reported as 32.67% by Li et al.^24^, 33.2% by Neugebauer et al.^25^ and 58% by Orhan et al.^26^. The rate of 37% in this study performed in the Turkish population is compatible with other studies in the literature. Regarding the effect of the presence of septa on sinus pathologies, no significant relationship was observed and this result is similar to the study of Taşssöker^27^ and Kocak^28^ who used the same imaging method.

Nasal septum deviation (NSD) is the displacement of the nasal septum, which is normally located in the midline of the face, to the left or right. NSD disrupts sinus drainage by narrowing the middle meatus and decreasing nasal air passage. Avsever et al.^29^ found the incidence of NSD to be 13.1% and Kaya et al.^28^ found it to be 89.7%. Many studies have examined the relationship between NSD and sinus pathologies^27,30–34^ Poorey^31^ and Taghiloo^32^ observed a significant correlation, while Taşsoker^27^, Kaya^30^, Balikci^33^ and Köse^34^ did not observe a correlation. In this study, NSD was found to be 54.1% and there was no significant correlation with sinus pathologies.

The paranasal sinuses are normally lined with 1 mm thick respiratory epithelium. It has been reported that the mucosa may thicken up to 10–15 times the normal thickness in case of inflammation. Some authors have accepted ≥ 1 mm, ≥ 2 mm, > 3 mm mucosal thickening as pathologic^13,35,36^. The prominent features of inflammatory sinus diseases are mucosal thickening, opacification and air-fluid level in the sinus^20^. In one study, mucosal thickening between 2 and 5 mm was found in 24.3%^37^. In this study, mucosal thickening above 2 mm was accepted as pathologic and 21.8% of them were found to be compatible with previous studies. When the factors causing mucosal thickening are eliminated with appropriate treatments before implant applications and sinus membrane perforations are repaired, there will be no risk for implant and graft applications^38^.

Mucous retention cysts are radiopacities which are usually asymptomatic and incidentally observed on radiographs extending dome-shaped from the sinus wall due to obstruction of the mucus-secreting glands of the maxillary sinus. Since mucous retention cysts regress spontaneously in most cases or do not cause serious volume change, follow-up is recommended if they do not lead to any complications^10,39^. The prevalence of mucous retention cysts has been evaluated in various studies; Aghaee et al.^39^ found 5.6%, Gracco et al.^35^ 5.75%, and Phothikhun et al.^40^ 10%. In the Turkish subpopulation we studied, this value was 12.3%. In this study, it was seen that the wall most affected by pathologies was significantly inferior. The wall to which retention cysts are attached will be important when sinus floor elevation technique will be applied, which makes our study valuable. This study will guide maxillofacial surgeons and otolaryngologists.

With the change in the air-fluid level in the maxillary sinus, the normally radiolucent maxillary sinus will appear more radiopaque^20^. In addition to Lana et al.^20^ who found partial or total opacification in the maxillary sinus at a rate of 1.8%, Rege et al.^41^ reported this rate as 7.8% and Raghav et al.^42^ reported this rate as 16.6%. In our study, this value was compatible with the literature with 5.1%.

The ostium has a major role in the drainage and ventilation of the maxillary sinus. When the maxillary ostium is obstructed, the amount of oxygen required to maintain metabolic activity of the sinus mucosa decreases. Thus, with the increase in anaerobic reactions, the necessary environment for bacterial proliferation is created^43^. This leads to mucosal thickening and increased radiopacity associated with inflammation and edema in the mucosa. Dobele et al.^44^ found the prevalence of ostium obstruction to be 26.5% in their study and stated that the relationship with mucosal thickening was significant. Carmeli et al.^45^, who had similar results with this study, calculated the prevalence of ostium obstruction as 15% and observed mucosal thickening in 65.48% and opacification in 34.52% of patients with obstruction. In this study, 39.6% of patients with ostium obstruction (10.6%) had opacification in the sinus, while 38.68% had mucosal thickening.

The close relationship of the maxillary posterior tooth roots with the sinus may cause pathogenic microorganisms and toxins in the oral cavity to reach the sinus through the spongiose bone and cause inflammation of the mucosa and sinusitis symptoms^46,47^. Kuligowski et al.^47^ who reached a similar conclusion with this study, found that mucosal thickening was higher in maxillary sinuses associated with tooth roots.

The limitation of this study was that the pediatric population could not be evaluated because only young and adult patients were included in the study. In addition, the medicine history of the patients could not be accessed. Future studies can be performed in a multicenter and larger sample to represent the general population.

## Conclusion

CBCT, a three-dimensional method, can be used to evaluate anatomical and pathological changes in the maxillofacial region. Pneumatization was the most common anatomical variation while mucous retention cyst was the most common sinus pathology. Sinus pathologies were particularly associated with pneumatization, sinus-associated tooth roots and obstruction of the ostium. Before dental implant and sinus surgery applications, detecting the presence of variations and pathologies can reduce complications such as sinus membrane perforation, infection, failure to break the bone window due to the presence of antral septa, graft loss and oroantral fistula formation. Otorhinolaryngologist consultation may be required if symptoms of sinusitis are present as opposed to being asymptomatic.

## Data Availability

If there is a valid reason, data can be obtained by contacting the corresponding author.
