# Author Correction: Retrospective analysis of pathological changes in the maxillary sinus with CBCT

**DOI:** 10.1038/s41598-024-69007-0

**Published:** 2024-08-06

**Authors:** Mehmet Emin Dogan, Nurbanu Uluısık, Semahat Doğru Yuvarlakbaş

**Affiliations:** 1 Faculty of Dentistry, Department of Dentomaxillofacial Radiology, Harran University, Sanliurfa, Turkey; 2 Faculty of Medicine, Department of Anatomy, Harran University, Sanliurfa, Turkey

Correction to: *Scientific Reports* 10.1038/s41598-024-66527-7, published online 05 July 2024

The original version of this Article contained an error in Table 3, where the *P* value ‘0.000*’ was incorrectly given as ‘1.000*’.

The original Article has been corrected.

